# Unique Regulation of Sed-1 β-Lactamase in *Citrobacter sedlakii*: Insights on Resistance to Third-Generation Cephalosporin

**DOI:** 10.3390/antibiotics14080823

**Published:** 2025-08-12

**Authors:** Mako Watanabe, Ryuichi Nakano, Keizo Yamamoto, Akiyo Nakano, Yuki Suzuki, Kai Saito, Satoko Nakashima, Kentaro Endo, Kazuya Narita, Hisakazu Yano

**Affiliations:** 1 Department of Microbiology and Infectious Diseases, Nara Medical University, 840 Shijo-cho, Kashihara 6348521, Nara, Japan; 2 Department of Chemistry, Nara Medical University, 88 Shijo-cho, Kashihara 6340813, Nara, Japan; 3 Division of Central Clinical Laboratory, Iwate Medical University Hospital, 1-1-1 Idaidori, Yahaba-cho, Shiwa-gun 0283694, Iwate, Japan

**Keywords:** *Citrobacter*, Sed-1 β-lactamase, regulation, expression, ESBL, mutant

## Abstract

**Background:** The *Citrobacter* genus harbors class C (AmpC) and class A β-lactamases. *Citrobacter freundii* produces an inducible AmpC β-lactamase controlled by the LysR-type transcriptional regulator AmpR and cytosolic amidase AmpD. *Citrobacter sedlakii* produces the class A β-lactamase Sed-1, whose expression is believed to be regulated by the transcriptional regulator SedR and AmpD. **Objectives:** *C. sedlakii* NR2807, isolated in Japan, is resistant to third-generation cephalosporins and displays extended-spectrum β-lactamase characteristics. Here, we sought to understand the mechanism for successful resistance to third-generation cephalosporins by investigating the regulators controlling Sed-1 production. **Methods:** Plasmids containing *bla*_Sed-1_ and *sedR* (pCR2807) or truncated *sedR* (pCR2807ΔSedR) were constructed and introduced into *Escherichia coli*. Antibiotic-resistant mutants of NR2807 were obtained, and enzyme kinetics were assessed. **Results:** The AmpD mutant (pCR2807/ML4953) showed an 8-fold increase in cefotaxime MIC and an 8.46-fold increase in Sed-1 activity compared to the wild-type (pCR2807/ML4947). However, induction of pCR2807/ML4947 also led to a 1.32-fold higher Sed-1 activity, indicating semi-inducibility. Deletion of *sedR* (pCR2807ΔSedR/ML4947) led to a 4-fold decrease in cefotaxime MIC and 1.93-fold lower Sed-1 activity, confirming SedR as an activator. While wild-type *C. sedlakii* ATCC51115 is susceptible to third-generation cephalosporins, the AmpD mutation in NR2807 led to Sed-1 overproduction and resistance to this class of antibiotics. Finally, mutagenesis revealed that amino acid substitution in Sed-1 conferred resistance to ceftazidime and extended-spectrum β-lactamase characteristics. **Conclusions:** Sed-1 producers, though usually susceptible to third-generation cephalosporins, may develop extended-spectrum β-lactamase traits due to AmpD or Sed-1 mutations, thereby requiring careful monitoring.

## 1. Introduction

Antimicrobial resistance represents a significant global public health concern, with β-lactamase-producing bacteria undermining the effectiveness of β-lactam antibiotics [1,2]. Among Enterobacterales, extended-spectrum β-lactamases (ESBLs) have drawn significant attention due to their ability to hydrolyze third-generation cephalosporins (3GC), thereby limiting the scope of critical antibiotics used in clinical settings [3]. β-Lactamases vary among *Citrobacter* species: *Citrobacter freundii* produces a class C β-lactamase (AmpC); whereas *Citrobacter amalonaticus*, *Citrobacter gillenii*, *Citrobacter koseri*, and *Citrobacter sedlakii* produce class A β-lactamases, CdiA, CKO-1, and Sed-1, respectively (Table 1) [4,5,6,7].

**Figure 1:**
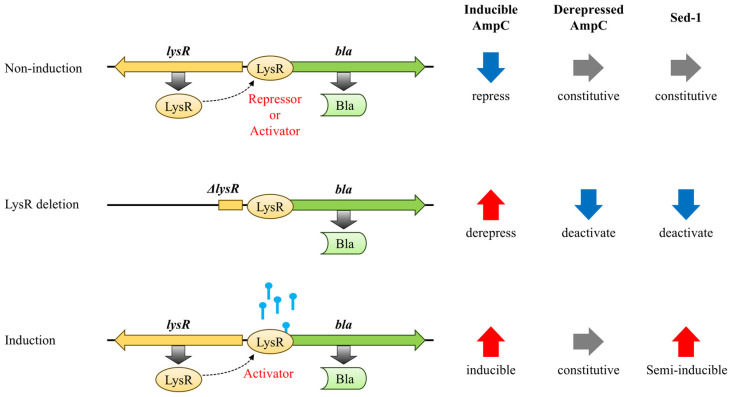
Schematic representation of Sed-1 and AmpC β-lactamase regulation. Inducible AmpC producers are repressed by AmpR but increase production when induced. Deletion of the *ampR* gene results in slightly higher basal expression. Derepressed AmpC producers are constitutively hyperproducing owing to mutated AmpR (Asp135Asn) with or without induction. Deletion of *ampR* results in lower expression. Sed-1 producers are semi-inducible, mirroring inducible AmpC producers. Deletion of the *sedR* gene results in reduced expression, akin to derepressed AmpC producers.

Gram-negative *C. freundii* expresses an inducible AmpC β-lactamase, which is regulated by the LysR-type transcriptional regulator *ampR* and the cytosolic N-acetylmuramyl L-alanine amidase AmpD [8,9,10]. Induction of AmpC involves the conversion of *ampR* from acting as a repressor—in the absence of an inducer—to becoming an activator. Certain AmpR mutants act as constant activators, causing constitutive overproduction of AmpC, regardless of the presence of an inducer (Figure 1, Supplementary Table S1) [8]. Specifically, the Asp135Asn AmpR substitution correlates with substantially higher β-lactamase activity in several Gram-negative bacteria, including *C. freundii*, *Enterobacter cloacae* complex, and *Pseudomonas aeruginosa* (Supplementary Figure S1) [11,12,13,14]. Mutations that permanently inactivate AmpD induce and increase muropeptide content in the cytoplasm and change the conformation of AmpR, repurposing it into a transcriptional activator [15,16]. Constitutive production of AmpC due to mutations in *ampR* or *ampD* is linked to successful resistance to 3GC [9,15].

In 2001, *C. sedlakii*, a member of the *C. freundii* complex, was first reported to harbor a chromosomally encoded Sed-1 (Table 1) [17]. This enzyme is associated with intrinsic resistance to narrow-spectrum β-lactams such as penicillins and narrow-spectrum cephalosporins. The LysR-type transcriptional regulator *sedR* is located upstream of *bla*_Sed-1_. Similarly to AmpC, Sed-1 expression is thought to be regulated by *sedR* and *ampD*. However, the regulatory mechanism and strong resistance exhibited by Sed-1-producing bacteria remain poorly understood owing to limited reports of *C. sedlakii* infections. This is partly due to the difficulty of identifying *C. sedlakii* isolates using standard automated bacterial identification systems [18]. Understanding the genetic and functional characteristics of Sed-1 is essential for elucidating its role in β-lactam resistance and potential clinical implications.

Here, we sought to understand the mechanism for successful resistance to 3GC by investigating the regulators affecting Sed-1 production. To this end, we focused on *C. sedlakii* NR2807, which exhibited resistance to 3GC, a characteristic of ESBL-producing bacteria, and *C. sedlakii* ATCC51115, a 3GC-sensitive reference strain. The objectives of this study were to: (1) confirm the taxonomic identity of NR2807 using various molecular methods; (2) evaluate the genetic and phenotypic features of *C. sedlakii* strains; (3) characterize genetic variations in *bla*_Sed-1_, *sedR*, and *ampD*; and (4) investigate the regulatory role of *sedR* and *ampD* in β-lactamase expression. Additionally, we aimed to determine the functional impact of mutations in *bla*_Sed-1_ on cephalosporin hydrolysis and substrate specificity by analyzing kinetic parameters.

To facilitate comprehension of specialized terminology used throughout this manuscript, a glossary of technical terms has been provided in the Supplementary Materials (Supplementary Table S2).

## 2. Results

### 2.1. Identification of C. sedlakii NR2807

Microscan Walkaway showed that NR2807 was most similar to *Citrobacter farmeri* (93.15%). Matrix-assisted laser desorption/ionization-time of flight mass spectrometry (MALDI-TOF MS) identified NR2807 as *C. amalonaticus* with a similarity of 99.9%. Comparison of *C. sedlakii* ATCC51115, *C. amalonaticus* FDAARGOS_1489, *C. farmeri* FDAARGOS 1423, *C. freundii* ATCC 8090, *C. koseri* FDAARGOS_86, *Citrobacter rodentium* NBRC 105723, *Citrobacter werkmanii* FDAARGOS_1524, *Citrobacter youngae* NCTC13709, and *Citrobacter braakii* FDAARGOS 1421 yielded average nucleotide identity (ANI) values of 98.88%, 84.13%, 83.86%, 81.26%, 82.51%, 86.07%, 81.25%, 81.12%, and 81.21%, respectively. Therefore, NR2807 was identified as *C. sedlakii* and was newly registered as ST1320 (*aspC* 400, *clpX* 422, *fadD* 274, *mdh* 205, *arcA* 237, *dnaG* 196, and *lysP* 226).

### 2.2. Antimicrobial Susceptibility and Resistance Gene

NR2807 was resistant to cefotaxime (16 µg/mL) but was inhibited by clavulanic acid (0.5 µg/mL), showing a pattern typical of ESBL-producing strains (Table 2). *C. sedlakii* ATCC51115 was resistant to ampicillin (>256 µg/mL) but was susceptible to cefotaxime (0.5 µg/mL) and ceftazidime (2 µg/mL), which is typical for class A β-lactamase-producing strains. NR2807 showed higher MIC for β-lactams and 65.47-fold higher β-lactamase activity compared to ATCC51115. Both were susceptible to cefepime, carbapenems, levofloxacin (0.125 µg/mL), and gentamicin (1 µg/mL).

Whole-genome analysis with ResFinder revealed that NR2807 and ATCC51115 harbored *bla*_Sed-1_ as the only resistance gene, with *sedR* located upstream of it in the opposite direction. Sed-1 (295 amino acid residues) from NR2807 differs from that of ATCC51115 by two amino acid substitutions (Asp175Gly and Arg228His); whereas SedR (286 amino acid residues) differs by a single amino acid substitution (Thr198Ala) and AmpD (187 amino acid residues) by four amino acid substitutions (Thr50Ala, Arg101His, Asp110Val, and Gln131Leu).

### 2.3. Effect of Regulator Genes on β-Lactamase Expression

To evaluate the effect of *sedR* and *ampD* on Sed-1 production, MIC and β-lactamase activity of Sed-1-producing transformants with wild-type or mutant AmpD were measured in the presence or absence of SedR. β-lactamase activity of the wild-type (pCR2807/ML4947) was 2.78 U/mg and it increased by 1.32-fold when induced by cefoxitin (Table 2); however, that of the AmpD mutant (pCR2807/ML4953) was 8.46-fold higher, reaching 23.50 U/mg. The MIC for cefotaxime was 8-fold higher in pCR2807/ML4953 than in pCR2807/ML4947. These findings indicate that Sed-1 was upregulated by SedR and AmpD. pCR2807ΔSedR/ML4947 showed a 4-fold lower MIC for cefotaxime and 1.93-fold lower β-lactamase activity compared to pCR2807/ML4947. In this case, SedR was not repressing Sed-1, but instead acted as a positive regulator both in the absence and especially in the presence of a β-lactam inducer.

**Figure 2:**
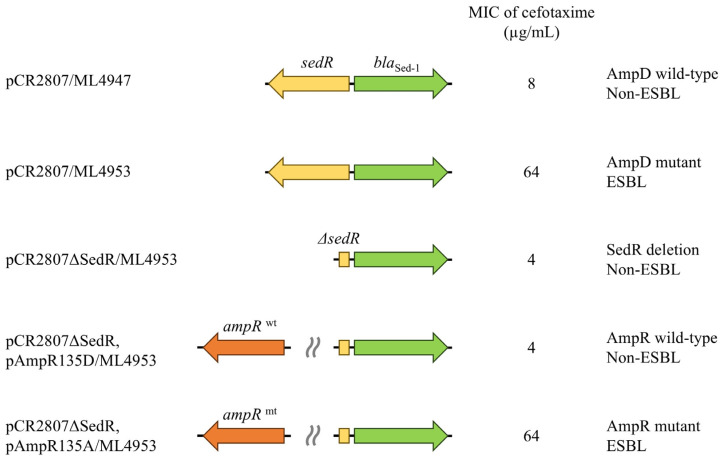
Altered drug sensitivity of Sed-1β-lactamase producers is attributed to mutations in regulatory genes. The AmpD mutant strain (pCR2807/ML4953) shows increased expression of Sed-1 and a higher MIC for cefotaxime (64 µg/mL), whereas *sedR* deletion (pCR2807ΔSedR/ML4953) results in decreased expression and a lower MIC (4 µg/mL). Co-expression of mutant AmpR in the *sedR*-deficient strain restores the MIC to 64 µg/mL.

To further elucidate the role of SedR in Sed-1 production, a plasmid carrying *ampR* was transformed into the *sedR*-deficient strain (pCR2807ΔSedR/ML4953) (Figure 2). Wild-type AmpR (pAmpR135D), derived from classical AmpC producers, functions as a repressor; whereas mutant AmpR (pAmpR135A) is in a derepressed (active) form. The wild-type *ampR* strain (pCR2807ΔSedR, pAmpR135D/ML4953) showed 1.66-fold lower Sed-1 activity (2.07 U/mg) compared to pCR2807ΔSedR/ML4953 (3.42 U/mg) (Table 2). In contrast, the mutant *ampR* strain (pCR2807ΔSedR, pAmpR135A/ML4953) showed 5.87-fold higher Sed-1 activity (20.08 U/mg) and a MIC for 3GC comparable to that of pCR2807/ML4953. These features were confirmed in ATCC51115 transformants. Therefore, SedR functions similarly to the AmpR mutant.

### 2.4. Characteristics of Various Antibiotic-Resistant Mutants

To investigate the mechanism of successful antibiotic resistance by Sed-1, strains with increased MICs for antibiotics used for selection were analyzed. The antibiotic-resistant mutants appeared at a frequency of ~10^−6^–10^−7^ for ceftazidime, cefepime, cefmetazole, aztreonam, and imipenem supplied at 2× or 4× MIC. Ten colonies of the mutants were randomly selected for each condition, and the *bla*_Sed-1_, *sedR*, and *ampD* genes were sequenced and compared to those of the parent *C. sedlakii* NR2807 strain. Sequence data revealed that only *bla*_Sed-1_ was altered upon ceftazidime selection. The mutant strains isolated on ceftazidime were found to possess three different sets of mutations in Sed-1 (Pro167Gln; Asp179Gly; and Ile173Met, Pro174Ala, 174_175insS) and drug sensitivity as listed in Table 3. Compared with the NR2807 parent strain, the MIC for ceftazidime was 16-fold higher, while the MIC for cefotaxime was 4- to 8-fold lower in the mutants. Strain NR4574 (Asp179Gly) also displayed a 4-fold lower MIC for aztreonam. Strain NR4575 (Ile173Met, Pro174Ala, 174_175insS) presented a 4-fold lower MIC for piperacillin. Each mutant strain was assumed to possess altered substrate specificity due to amino acid substitutions. To evaluate whether the higher MIC in ceftazidime-resistant mutants was due to mutations in Sed-1, *bla*_Sed-1_, and *sedR* of strain NR4573 (Pro167Gln) were cloned into *Escherichia coli* ML4947 and compared with wild-type transformant pCR2807/ML4947. In line with the original NR4573 strain, pCR4573/ML4947 displayed higher ceftazidime MIC and lower cefotaxime MIC.

No mutations in *bla*_Sed-1_, *sedR*, and *ampD* were found in the other antibiotic-selected mutants. Mutant strain NR4584 selected on 0.5 µg/mL imipenem showed an 8-fold higher MIC for the antibiotics used for selection. Mutant strain NR4062 selected on 8 µg/mL cefmetazole showed 16-fold higher MIC for the antibiotics used for selection, 4-fold higher MIC for cefepime, and 32-fold higher MIC for cefotaxime combined with clavulanic acid. Mutant strains NR4586 and NR5701 selected on 8 µg/mL cefepime and 256 µg/mL aztreonam, respectively, exhibited 4-fold higher MIC for the antibiotic used for selection, along with 8- and 4-fold higher MIC for cefotaxime. Genomic analysis of one representative of each antibiotic mutant strain revealed mutations in various genes (Table 3). Selection with cefepime, aztreonam, and imipenem yielded mutations in *cdsA*, a phosphatidic acid cytidyl transferase; whereas cefmetazole selection yielded mutations in *rseA*, an anti-sigma-E factor.

### 2.5. Kinetic Parameters of Ceftazidime-Resistant Mutants

To evaluate the substrate specificity of wild-type Sed-1 and its ceftazidime-resistant mutants, kinetic parameters were measured for *E. coli* BL21 transformants harboring wild-type *bla*_Sed-1_ (NR2807), and ceftazidime-resistant-mutant *bla*_Sed-1_ (NR4573, NR4574, and NR4575) (Table 4). NR2807 showed lower catalytic efficiency (*k*_cat_/*K*_m_ = 1.31 s^−1^ mM^−1^) against ceftazidime, while NR4573 (Pro167Gln), NR4574 (Asp179Gly), and NR4575 (Ile173Met, Pro174Ala, 174_175insS) showed 4.24-, 11.60-, and 30.92-fold higher hydrolytic activity (*k*_cat_/*K*_m_) than NR2807. On the one hand, *K_m_* values were more than 17.78-fold lower in the mutants than in wild-type Sed-1, resulting in substantially higher *k*_cat_/*K_m_*. On the other hand, they showed 2.11-, 21.83-, and 1.95-fold lower hydrolytic activity against cefotaxime than NR2807, respectively.

## 3. Discussion

### 3.1. Genomic and Clinical Significance of Sed-1 in C. Sedlakii

Sed-1 β-lactamase was reported in 2001 as a chromosomally encoded class A β-lactamase in *C. sedlakii* [17]. Few reports on *C. sedlakii* isolates followed, probably because the automated bacterial identification systems commonly used in clinical practice failed to identify this species. In this study, Gram-negative rods were identified via whole-genome ANI analysis as *C. sedlakii* strain NR2807. To the best of our knowledge, this is the first reported clinical isolation of a Sed-1 producer in Japan. According to PubMLST (https://pubmlst.org/bigsdb?db=pubmlst_cfreundii_isolates, accessed on 6 August 2025), only 13 types of STs have been attributed to *C. sedlakii*, including ST1320, which was newly registered in this study.

Whole-genome analysis showed that *bla*_Sed-1_ was the only chromosomally encoded β-lactamase in NR2807. As in the type strain ATCC51115, it coded for the regulatory gene *sedR*. Sed-1 in NR2807 differed from its counterpart in ATCC51115 by amino acid substitutions Asp175Gly and Arg228His. These substitutions have not been related to extended changes in substrate specificity of class A β-lactamases TEM and SHV [19]. Indeed, *E. coli* transformants (pCR2807/ML4947 and pCR51115/ML4947) harboring these β-lactamases showed similar MICs.

### 3.2. Functional Impact of AmpD Mutation on Sed-1 Expression and Antibiotic Resistance

The MIC of NR2807 revealed resistance to a broad range of β-lactams, including 3GC, as well as ESBL-like characteristics; whereas ATCC51115 had an overall lower MIC and no ESBL-like properties (Table 2). Sed-1 production was 65.47-fold higher in NR2807 than in ATCC51115, which may have a significant impact on antimicrobial susceptibility. To elucidate the mechanism regulating Sed-1 expression, *bla*_Sed-1_–*sedR* was cloned into *E. coli* with or without the AmpD mutation, and the effect on MIC and enzyme production was assessed. Enzyme activity experiments revealed that pCR2807/ML4947 (AmpD wild-type) and pCR2807/ML4953 (AmpD mutant) increased Sed-1 production by 1.32- and 1.56-fold following induction. Without induction, Sed-1 output was 8.46-fold higher in pCR2807/ML4953 than in pCR2807/ML4957, and the same trend was observed for MIC, suggesting that increased Sed-1 resulted in higher MIC. Accordingly, SedR causes only partial induction of Sed-1, rather than the strong inducibility typically observed in AmpR. Furthermore, Sed-1 expression was strongly influenced by AmpD mutations (Figure 1). In AmpC-producing bacteria, AmpD inactivation leads to the accumulation of cytoplasmic muropeptides, which bind to and activate the transcriptional regulator AmpR. A similar mechanism is likely at play in *C. sedlakii*, where accumulated muropeptides interact with SedR, shifting it from an inactive to an active transcriptional state. This suggests that SedR requires muropeptide binding to function as a transcriptional activator, and that AmpD mutations indirectly enhance Sed-1 expression through this activation pathway. Therefore, the AmpD mutant mimics an induced state, functionally equivalent to the “Induction” scenario depicted in Figure 1, resulting in elevated β-lactamase activity and increased resistance to 3GC.

### 3.3. SedR Functions as a Constitutive Activator Similar to Mutant AmpR

**Figure 3:**
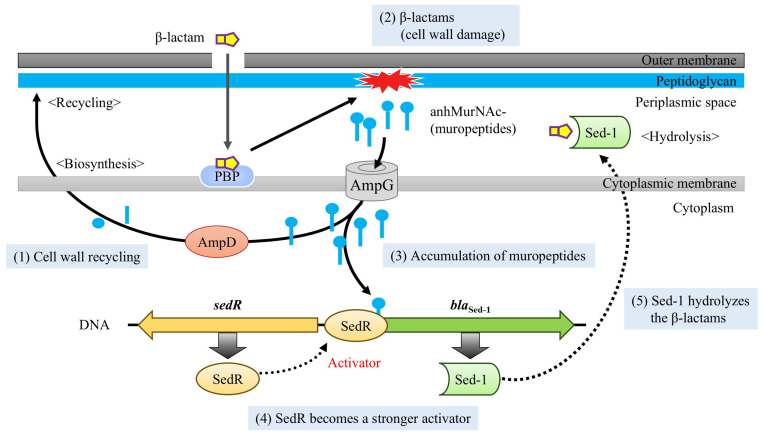
Schematic representation of Sed-1 β-lactamase regulation. (1) In the basal state, cell wall degradation products (muropeptides) enter the cytoplasm via AmpG and are processed by AmpD for biosynthesis or recycling. (2) When the cell wall is damaged by β-lactams, (3) muropeptides accumulate in the cytoplasm. (4) Upon interacting with muropeptides, SedR is converted into a stronger activator in a semi-inducible manner, promoting further production of Sed-1. (5) Sed-1 then hydrolyzes β-lactams in the periplasmic space.

The *sedR*-deficient strain (pCR2807ΔSedR/ML4947) displayed 1.93-fold lower Sed-1 production and lower MIC than the *sedR*-positive strain (pCR2807/ML4947). Deletion of *ampR* in inducible AmpC producers resulted in slightly more basal *ampC*, but no further induction of enzyme synthesis (Figure 1, Supplementary Table S1) [8,20]. In the AmpR (Asp135Ala) mutant, AmpR works as an activator regardless of the presence of an inducer, and its deletion lowers *ampC* expression [12]. Given that SedR functions similarly to the mutant AmpR, wild-type AmpR, and mutant AmpR were co-inserted in a *sedR*-deficient strain, and their effect on Sed-1 expression was analyzed. The Sed-1 producer (pCR2807/ML4953) shared the same MIC and enzyme activity as the mutant AmpR-carrying strain (pCR2807ΔSedR, pAmpR135D/ML4953) (Figure 2). Transcriptional regulators of the LysR family are known to act similarly on related genes [21,22]. This result indicates that SedR functions as an activator, akin to mutant AmpR, rather than as a repressor like wild-type AmpR (Figure 3). The amino acid in SedR corresponding to position 135 of AmpR is Asn (Supplementary Figure S1). The Asp135Asn substitution in AmpR has been shown to result in an active form in various bacterial species [8,11,13,14], further confirming the activator-like function of SedR (Supplementary Table S1). Hence, expression of *bla*_Sed-1_ is regulated by SedR, which acts like the AmpR mutant to activate Sed-1 in the absence of an inducer or aids its production in the presence of an inducer. This mechanism appears unique to Sed-1 compared to other β-lactamases (Figure 1).

ATCC51115 clone strains exhibited similar Sed-1 activity and MIC as NR2807 clone strains, indicating a similar expression mechanism influenced by SedR and AmpD. NR2807 has two amino acid substitutions in Sed-1 compared to ATCC51115, which may have an effect. However, increased Sed-1 production and MIC values were more likely caused by AmpD mutations than by amino acid substitutions. Therefore, NR2807 has become broadly and highly resistant due to the AmpD mutation, whereas ATCC51115 has low Sed-1 production and MIC.

### 3.4. Ceftazidime-Driven Mutations in Sed-1 Expand Substrate Specificity

To further elucidate the mechanism of β-lactamase expression in NR2807, experiments were conducted to generate a drug-resistant mutant strain. Similar experiments with AmpC producers, such as *C. freundii* and *E. cloacae*, yielded mutations in *ampD* and *ampR* [11,15,23], but no Sed-1-producing mutants with additional mutations in *ampD* or *sedR* could be isolated. These factors could explain the different mutation rates among bacterial species, the role of SedR as an inducible high-producing type, and NR2807 as an AmpD mutant [24]. Here, only the ceftazidime-resistant mutant strain showed an amino acid substitution in Sed-1, which likely expanded substrate specificity and, in this way—rather than via upregulation—improved resistance.

Among the amino acids involved in extended substrate specificity of class A β-lactamase mutants, single substitutions were identified in NR4573 (Pro167Gln) and NR4574 (Asp179Gly) [4,19]. The increased affinity of the mutants for ceftazidime (29.78- and 1213.94-fold decrease in *Km*, respectively) may have resulted in higher catalytic efficiency and increased MIC. Strain NR4575 had two amino acid substitutions and one insertion (Ile173Met, Pro174Ala, 174_175insS) not previously known to expand substrate specificity of class A β-lactamases. All Class A β-lactamases have a conserved structural feature, the omega loop, which spans residues 161 to 179 or 164 to 179 and forms part of the active site pocket [25,26,27]. The above amino acid substitutions are located in the omega-loop and likely cause a conformational change, thereby increasing enzyme activity against ceftazidime. Interestingly, Sed-1 mutants were obtained in ceftazidime only. No mutations were observed in the regulatory genes of other antibiotics, although mutations were observed in *cdsA* and *rseA* [28,29]. These genes are thought to be related to the acquisition of antibiotic resistance through extended substrate specificity.

### 3.5. ESBL-like Features and Future Risk in Sed-1-Producing C. Sedlakii

**Figure 4:**
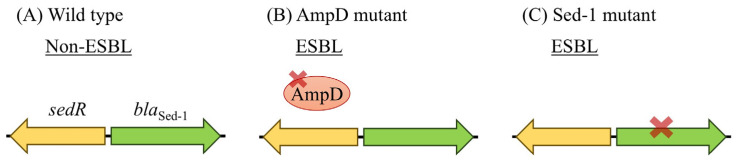
Schematic illustration of the evolution of Sed-1 β-lactamase activity in *C. sedlakii*. (**A**) In the wild-type strain, Sed-1 exhibits a narrow-spectrum profile and does not show ESBL properties. (**B**) In the presence of an AmpD mutation, Sed-1 expression is markedly increased, leading to ESBL-like resistance, particularly against third-generation cephalosporins (3GC). (**C**) Acquisition of Sed-1 structural mutations (e.g., in the omega-loop) can expand substrate specificity, also conferring ESBL-like properties.

In the first report on Sed-1 producing *C. sedlakii*, Sed-1 showed high catalytic efficiency against narrow-spectrum β-lactams, such as aminopenicillins, carboxypenicillins, and first-generation cephalosporins. Sed-1 producers were resistant to these compounds but were susceptible to 3GC and carbapenems [17]. The wild-type ATCC51115 has a low MIC for extended-spectrum cephalosporins and is not considered an ESBL (Figure 4). Instead, NR2807 exhibited ESBL-like characteristics, such as a high MIC for extended-spectrum cephalosporins. Given reports of *C. sedlakii* carrying the ESBL gene [18,30], any inquiry into resistance mechanisms by this species will require clarification of ESBLs but also regulatory genes. Furthermore, the catalytic efficiency and MIC exhibited by the ceftazidime mutants obtained in this study clearly suggested they were ESBL producers. Given a history of ESBL types in TEM- and SHV-type Class A β-lactamases, ESBL types may also emerge in Sed-1-producing *C. sedlakii* [31,32], requiring careful monitoring.

### 3.6. Study Limitations

This study has some limitations. First, the role of SedR in Sed-1 production is not fully understood. Both strains used in the analysis were active, but there may be mutations that result in even higher output, while others may be suppressive, as in inducible AmpC producers. To elucidate the regulatory mechanism employed by SedR, the function of mutants should be analyzed. Second, several mutations were identified in *C. sedlakii* strains selected for antibiotics other than ceftazidime, but the cause of resistance to the drug remains unclear. Cloning of each candidate gene would reveal whether its mutation caused increased resistance in Sed-1-producing bacteria. Finally, this study focused on only two strains; more strains need to be collected and analyzed in detail to fully understand the mechanism of Sed-1 production. As this study reveals the possible emergence of ESBL-type Sed-1 producers, such occurrence should be carefully monitored.

## 4. Materials and Methods

### 4.1. Bacterial Strains and Antimicrobial Susceptibility Testing

Glucose-fermenting Gram-negative rod-shaped NR2807 was isolated from a blood sample of a hospitalized patient in Japan in 2018. The species was identified by MicroScan WalkAway plus (Beckman Coulter, Inc., Lane Cove West, NSW, Australia), MALDI-TOF MS (VITEK^®^ MS; bioMérieux, North Ryde, NSW, Australia) and ANI analysis using whole-genome sequences (JSpeciesWS, Bremen, Germany) [33]. *C. sedlakii* ATCC51115 (NBRC105722) was used as the reference strain. To evaluate the effect of *sedR* and *ampD* on Sed-1 production, plasmids containing both *bla*_Sed-1_ and *sedR* (pCR2807) or *bla*_Sed-1_ and truncated *sedR* (pCR2807ΔSedR) were constructed using the Zero Blunt TOPO PCR Cloning Kit (Thermo Fisher Scientific, Waltham, MA, USA) with primers Sed-1down (5′-TGTCTGCGCAGGGTTCTGTTC-3′) and SedRdown (5′-TGGTACGCTGATCCCCGAAC-3′), and Sed-1down and SedRUpR (5′-CGGCGGGATGCGACAGT-3′), respectively. The plasmids were transformed into *E. coli* ML4947 (AmpD wild-type) and ML4953 (AmpD mutant) [34]. In vitro ceftazidime-resistant mutant strains were isolated from NR2807. The bacterial strains and plasmids used in this study are listed in Table 5. To evaluate the effect of LysR mutation on antimicrobial susceptibility and β-lactamase expression, *ampR* clone plasmids were transformed into *E. coli* harboring *bla*_Sed-1_ and truncated *sedR*. The *ampR* clone plasmids pAmpR135D and pAmpR135A used in this study were previously constructed from *C. freundii* (Supplementary Figure S1) [12].

Antibiotic susceptibility was determined using the agar dilution method according to Clinical and Laboratory Standards Institute guidelines [35].

### 4.2. Whole-Genome Sequencing and Analysis

Genomic DNA of NR2807 was extracted using QIAGEN Genomic-tip 500/G. Whole genomes were sequenced using MiSeq (Illumina Inc., San Diego, CA, USA) and MinION (Oxford Nanopore Technologies, Oxford, UK), after which they were subjected to hybrid de novo assembly using Unicycler v0.5.0 [36]. Species were identified based on ANI between strain NR2807 (GenBank accession no. BAAHNL01000000.1), *C. sedlakii* ATCC51115 (GenBank accession no. GCA_000759835.1), *C. amalonaticus* FDAARGOS_1489 (GenBank accession no. GCF_020099335.1), *C. farmeri* FDAARGOS 1423 (GenBank accession no. GCA_019048065.1), *C. freundii* ATCC 8090 (GenBank accession no. CP049015.1), *C. koseri* FDAARGOS_86 (GenBank accession no. GCA_ 000783445.2), *C. rodentium* NBRC 105723 (GenBank accession no. GCA_021278985.1), *C. werkmanii* FDAARGOS_1524 (GenBank accession no. GCA_ 020341495.1), *C. youngae* NCTC13709 (GenBank accession no. GCA_ 900638065.1), and *C. braakii* FDAARGOS 1421 (GenBank accession no. GCA_019048805.1). Earlier studies recommended an ANI of 95–96% as a species demarcation cut-off [37,38].

Antimicrobial resistance genes in the genome sequence were identified by searching the ResFinder database (http://genepi.food.dtu.dk/resfinder (accessed on 6 August 2025)) using thresholds of 90% identity and 60% minimum length [39]. Amino acid numbering of Sed-1 corresponded to the class A β-lactamase [40]. Multilocus sequence typing of *Citrobacter* spp. isolates was performed as previously described [41]. Sequence types were assigned by the PubMLST database (https://pubmlst.org/organisms/citrobacter-spp (accessed on 6 August 2025)).

### 4.3. Measurement of β-Lactamase Activity and Induction Assays

β-Lactamase activity was measured by a colorimetric assay as previously described [34,42]. Briefly, bacterial strains were cultured in Mueller-Hinton broth to mid-logarithmic phase with shaking at 37 °C, and the protein contents in crude extracts were suspended in 50 mM phosphate buffer (pH 7.0). Enzyme activity was determined at 30 °C using a spectrophotometer (UV-1900i Plus; Shimadzu, Kyoto, Japan) with cephalothin (262 nm) as substrate. One unit of β-lactamase activity was equivalent to the amount of β-lactamase that hydrolyzed 1 μmol of β-lactam in 1 min at 30 °C. Protein concentration was measured using the Bradford assay [43]. All experiments were repeated three times, and enzymatic activity was determined as U/mg of protein. For the induction assay, bacterial strains were cultured in Mueller-Hinton broth to mid-logarithmic phase and subjected to 1/16 × MIC for cefoxitin as the inducer for 1 h [11].

### 4.4. Selection of Antibiotic-Resistant Strains and Detection of Sequence Changes

Antibiotic-resistant mutants were obtained by plating ~10^9^ colony-forming units (CFU)/mL of late-logarithmic-phase NR2807 grown in Luria–Bertani (LB) broth on LB agar plates containing ceftazidime, cefotaxime, cefepime, cefmetazole, aztreonam, or imipenem at 2× or 4× MIC, as previously described [44]. The mutation frequency was determined by dividing the colony density in CFU/mL on LB agar plates containing the antibiotic by the total colony density in CFU/mL. DNA sequences of *bla*_sed-1_, *sedR*, and *ampD* of selected mutants were determined by Sanger sequencing. The primers used are listed in Supplementary Table S3 [17]. Nucleotides and amino acids of selected mutants were compared to those of NR2807. For strains without mutations in these genes, whole-genome sequencing using MiSeq and annotation using DFAST were performed as above. The obtained genome sequences were compared with the sequence of NR2807 to search for mutations.

### 4.5. Measurement of Kinetic Parameters in NR2807 and Mutants

Amplicons of NR2807 and mutants (NR4573, NR4574, and NR4575) between primers Sed-1F-atg (5′-CTTAAAGAACGGTTTCGCCAGAC-3′) and Sed-1R+BamHI (5′-ATATGGATCCTTACTTTCCTTCCGTCAC-3′) were generated using Q5 Hot Start High-Fidelity 2× Master Mix (New England Biolabs, Ipswich, MA, USA). The amplicons were purified using the High Pure PCR Cleanup Micro Kit (Roche) and digested with *Bam*HI. The resulting fragments underwent kination by T4 Polynucleotide Kinase (TaKaRa Bio, San Jose, CA, USA) and were ligated with a previously described pET28a vector (Merck Millipore, Burlington, MA, USA) [45] using Ligation High Ver. 2 (Toyobo, Osaka, Japan). The resulting plasmids were transformed into One Shot TOP10 Chemically Competent *E. coli* (Thermo Fisher Scientific). Then, the plasmids were isolated from the transformants using the High Pure Plasmid Isolation Kit (Roche, Basel, Switzerland) and transformed into *E. coli* BL21(DE3) (Thermo Fisher Scientific) by electroporation. The resulting strains were inoculated into LB broth supplemented with 30 µg/mL kanamycin and incubated at 37 °C. Isopropyl thio-β-D-galactoside (final concentration 0.02 mM) was added when the optical density at 600 nm reached 0.5, and the culture was further incubated for 20 h at 20 °C. Cells were resuspended in buffer A (20 mM Tris/HCl, pH 7.5) and sonicated. The cell-free extract was applied to a Macro-Prep High S Support column (Bio-Rad Laboratories, South Granville, NSW, Australia) and washed with buffer A. The enzymes were eluted with buffer A containing 0.3 M NaCl. Fractions containing Sed-1 or mutant enzymes were pooled, dialyzed against buffer A, and injected into a CM-Toyopearl 650S column (Tosoh, Tokyo, Japan) equilibrated with buffer A. The enzymes were eluted with a linear gradient of NaCl (0–0.25 M) in the same buffer. Enzyme activity was determined with a spectrophotometer (V-730 BIO; JASCO, Hachioji, Japan) at 30 °C in 20 mM HEPES buffer pH7.0. The wavelengths and extinction coefficients used in this study were the same as described previously [45,46]. Protein concentration was determined by Bradford assay [43]. The enzyme was diluted with assay buffer containing 20 µg/mL bovine serum albumin to prevent denaturation. The values of the kinetic parameters (*K*_m_ and *k*_cat_) were obtained by a double-reciprocal (Lineweaver-Burk) plot of initial steady-state velocities at different substrate concentrations.

### 4.6. Nucleotide Sequence Accession Numbers

Whole-genome DNA sequences of NR2807 were deposited in the GenBank database under accession no. BAAHNL01000000.1

## 5. Conclusions

In conclusion, the mechanism of Sed-1 production in *C. sedlakii* appears to differ from that of other β-lactamase-producing bacteria. SedR promotes Sed-1 expression even when not induced and partially promotes it when induced. In particular, the AmpD mutant led to strong expression of Sed-1 and, consequently, elevated resistance. The possibility of ESBL-type emergence in Sed-1 mutant strains has been noted and should be carefully monitored.

## Figures and Tables

**Table 1:** Chromosomal β-lactamases produced by the *Citrobacter* genus.

Species		β-Lactamase (Class)	Regulator Gene	Expression
*C. freundii* complex				
	*C. braakii*	AmpC (C)	AmpR	Inducible
	*C. freundii*	AmpC (C)	AmpR	Inducible
	*C. murliniae*	AmpC (C)	AmpR	Inducible
	*C. youngae*	AmpC (C)	AmpR	Inducible
	*C. werkmanii*	AmpC (C)	AmpR	Inducible
	*C. portucalensis*	AmpC (C)	AmpR	Inducible
	*C. gillenii*	GIL-1 (A)	-	Constitutive
	*C. sedlakii*	Sed-1 (A)	SedR	Inducible
*C. farmeri*		Sed-1 (A)	SedR	Inducible
*C. rodentium*		Sed-1 (A)	SedR	Inducible
*C. amalonaticus*		CdiA (A)	CdiR	Inducible
*C. koseri*		CKO-1 (A)	-	Constitutive
*C. cronae*		AmpC (C)	AmpR	Inducible
*C. pasteurii*		AmpC (C)	AmpR	Inducible

**Table 2:** Susceptibility and β-lactamase activity of *C. sedlakii* strains and their reconstructed transformants.

Species	Strain	Genes	AmpD	MIC (µg/mL) ^a^									Relative β-Lactamase Activity (U/mg Protein) ^c^		
				PIP	CTX	CTX/CLA ^b^	CAZ	FEP	CMZ	CFX	ATM	IPM	Basal	Induced ^d^	Induced/ Basal
*C. sedlakii*	NR2807	*bla*_Sed-1_, *sedR*	mutant	256	32	0.5	16	4	2	8	64	0.125	8.39	8.54	1.02
*C. sedlakii*	ATCC51115	*bla*_Sed-1_, *sedR*	wild-type	8	0.5	0.25	2	0.125	2	8	1	0.25	0.13	0.40	3.11
*E. coli*	pCR2807/ML4947	*bla*_Sed-1_, *sedR*	wild-type	256	8	≤0.06	4	1	1	4	16	0.125	2.78	3.68	1.32
*E. coli*	pCR2807/ML4953	*bla*_Sed-1_, *sedR*	mutant	>256	64	1	16	4	1	4	64	0.125	23.50	36.66	1.56
*E. coli*	pCR2807ΔSedR/ML4947	*bla*_Sed-1_	wild-type	64	2	≤0.06	2	0.25	1	4	8	0.125	1.44	NT ^e^	-
*E. coli*	pCR2807ΔSedR/ML4953	*bla*_Sed-1_	mutant	256	4	≤0.06	2	0.5	2	4	8	0.125	3.42	NT	-
*E. coli*	pCR2807ΔSedR, pAmpR135D/ML4953	*bla*_Sed-1_, *ampR*^wt^	mutant	64	4	≤0.06	4	1	2	4	16	0.25	2.07	6.15	2.98
*E. coli*	pCR2807ΔSedR, pAmpR135A/ML4953	*bla*_Sed-1_, *ampR*^mt^	mutant	>256	64	0.125	16	4	1	4	64	0.25	20.08	23.50	1.17
*E. coli*	pCR51115/ML4947	*bla*_Sed-1_, *sedR*	wild-type	128	4	≤0.06	4	1	2	4	16	0.25	2.40	3.79	1.58
*E. coli*	pCR51115/ML4953	*bla*_Sed-1_, *sedR*	mutant	256	16	0.5	16	4	1	4	64	0.25	16.39	22.41	1.37
*E. coli*	pCR51115ΔSedR/ML4947	*bla*_Sed-1_	wild-type	64	2	≤0.06	2	0.25	1	4	8	0.25	1.48	NT	-
*E. coli*	pCR51115ΔSedR/ML4953	*bla*_Sed-1_	mutant	256	2	≤0.06	2	0.5	1	4	8	0.125	3.35	NT	-
*E. coli*	pCR51115ΔSedR, pAmpR135D/ML4953	*bla*_Sed-1_, *ampR*^wt^	mutant	64	2	≤0.06	4	1	1	2	8	0.25	2.85	4.14	1.45
*E. coli*	pCR51115ΔSedR, pAmpR135A/ML4953	*bla*_Sed-1_, *ampR*^mt^	mutant	>256	32	≤0.06	16	4	1	2	32	0.25	14.85	16.71	1.13
*E. coli*	ML4947	-	wild-type	2	≤0.06	≤0.06	0.25	≤0.06	1	0.5	≤0.06	≤0.06	<0.01	NT	-
*E. coli*	ML4953	-	mutant	4	≤0.06	≤0.06	0.5	≤0.06	1	0.5	≤0.06	≤0.06	<0.01	NT	-

^a^ Antibiotics: PIP, piperacillin; CTX, cefotaxime; CLA, clavulanic acid; CAZ, ceftazidime; FEP, cefepime; CMZ, cefmetazole; CFX, cefoxitin; ATM, aztreonam; IPM, imipenem. ^b^ MICs were determined in the presence of clavulanic acid (5 µg/mL). ^c^ β-Lactamase activities are the geometric mean values of three independent cultures. The standard deviations were within 10%. ^d^ 1/16× MIC of cefoxitin was used as the inducer. ^e^ NT, Not tested.

**Table 3:** Mutations and MICs of *C. sedlakii* NR2807 mutants.

Species	Strains	Selection ^a,b^	Mutation ^c^				MIC (µg/mL) ^a^								
			Sed-1 ^d^	SedR	AmpD	Other Mutated Genes	PIP	CTX	CTX/CLA ^e^	CAZ	FEP	CMZ	CFX	ATM	IPM
*C. sedlakii*	NR2807	-	-	-	-	-	256	32	0.5	16	4	2	8	64	0.125
*C. sedlakii*	NR4573	CAZ 64	P167Q	-	-	-	128	8	0.25	256	1	2	8	128	0.125
*C. sedlakii*	NR4574	CAZ 64	D179G	-	-	-	128	4	0.25	>256	1	2	8	16	0.125
*C. sedlakii*	NR4575	CAZ 64	I173M, P174A, 174_175insS	-	-	-	64	8	0.25	>256	2	2	8	32	0.125
*C. sedlakii*	NR4586	FEP 8	-	-	-	*citC*, *cdsA*, *ispH*	>256	256	0.25	16	16	2	4	256	0.5
*C. sedlakii*	NR4062	CMZ 8	-	-	-	*rseA*	256	64	16	16	16	32	64	128	0.25
*C. sedlakii*	NR5701	ATM 256	-	-	-	*tsuA*, *cdsA*, *ubiD*	>256	128	0.25	16	8	1	4	256	0.5
*C. sedlakii*	NR4584	IPM 0.5	-	-	-	*pbpA*, *cdsA*	256	32	0.5	16	4	2	8	64	1
*E. coli*	pCR4573/ML4947	-	P167Q	-	-	-	64	2	≤0.06	64	0.5	1	4	16	0.125
*E. coli*	pCR2807/ML4947	-	-	-	-	-	256	8	≤0.06	4	1	4	4	16	0.125

^a^ Antibiotics: PIP, piperacillin; CTX, cefotaxime; CLA, clavulanic acid; CAZ, ceftazidime; FEP, cefepime; CMZ, cefmetazole; CFX, cefoxitin; ATM, aztreonam; IPM, imipenem. ^b^ Numbers indicate the selected antibiotics concentration (µg/mL). ^c^ Abbreviations; P, Pro; Q, Gln; D, Asp; G, Gly; I, Ile; M, Met; A, Ala; ins, insertion; *citC*, [Citrate [pro-3S]-lyase] ligase; *cdsA*, phosphatidate cytidylyltransferase; *ispH*, 4-hydroxy-3-methylbut-2-enyl diphosphate reductase; *rseA*, anti-sigma-E factor; *pbpA*, penicillin-binding protein 2; *tsuA*, thiosulfate utilization transporter; *ubiD*, 3-octaprenyl-4-hydroxybenzoate carboxy-lyase. ^d^ Amino acid numbering corresponds to class A β-lactamase (40). ^e^ MICs were determined in the presence of clavulanic acid (5 µg/mL).

**Table 4:** Kinetic parameters of ceftazidime-resistant mutants of *C. sedlakii* NR2807.

β-Lactamase and Parameter	Value for Antibiotic ^a^			
	Piperacillin	Cefotaxime	Ceftazidime	Aztreonam ^b,c^
Wild-type (NR2807)				
*k*_cat_ (s^−1^)	624 ± 31.3	230 ± 21.3	4.55 ± 0.612	9.26 ± 0.133
*K*_m_ (μM)	326 ± 29.5	264 ± 30.2	3484 ± 486	29.6 ± 2.65
*k*_cat_/*K*_m_ (s^−1^·mM^−1^)	1910	871	1.31	313
P167Q mutant (NR4573)				
*k*_cat_ (s^−1^)	163 ± 1.24	172 ± 3.12	0.649 ± 0.0542	4.42 ± 0.0496
*K*_m_ (μM)	43.0 ± 1.56	417 ± 9.19	117 ± 11.9	55.1 ± 2.77
*k*_cat_/*K*_m_ (s^−1^·mM^−1^)	3790	413	5.55	80.2
D179G mutant (NR4574)				
*k*_cat_ (s^−1^)	2.57 ± 0.0551	0.213 ± 0.0017	0.0436 ± 0.0013	NH
*K*_m_ (μM)	10.5 ± 1.22	5.34 ± 0.273	2.87 ± 0.46	NH
*k*_cat_/*K*_m_ (s^−1^·mM^−1^)	245	39.9	15.2	ND
I173M, P174A, 174_175insS mutant (NR4575)				
*k*_cat_ (s^−1^)	62.5 ± 2.13	6.06 ± 0.262	7.94 ± 0.862	NH
*K*_m_ (μM)	84.0 ± 6.55	13.6 ± 1.89	196 ± 24.1	NH
*k*_cat_/*K*_m_ (s^−1^·mM^−1^)	744	446	40.5	ND

^a^ *K*_m_ and *k*_cat_ were calculated as means ± SD from three independent experiments. ^b^ NH, no hydrolysis was detected. ^c^ ND, not determined.

**Table 5:** Bacterial strains and plasmids used in this study.

Bacterial Strains or Plasmids	Characteristics ^a^
Strains	
NR2807	Clinical isolate of *C. sedlakii* from Japan
ATCC51115	Reference strain of *C. sedlakii* purchased from NITE Biological Resource Center
NR4573	Ceftazidime-resistant mutant of *C. sedlakii* NR2807 with Sed-1 mutation (P167Q)
NR4574	Ceftazidime-resistant mutant of *C. sedlakii* NR2807 with Sed-1 mutation (D179G)
NR4575	Ceftazidime-resistant mutant of *C. sedlakii* NR2807 with Sed-1 mutation (I173M, P174A, 174_175insS)
ML4947	*E. coli* (F^−^ *galK2 galT22 hsdR hsdM lacY1 metB1 relA supE44* Rif ^r^), cloning host with AmpD wild type
ML4953	*E. coli* (F^−^ *galK2 galT22 hsdR hsdM lacY1 metB1 relA supE44* Rif ^r^ *ampD9*), cloning host with AmpD mutant
TOP10	*E. coli* (F^−^ *mcrA* Δ(*mrr-hsdRMS-mcrBC*) φ80*lacZ*ΔM15 Δ*lacX*74 *nupG recA1 araD139* Δ(*ara leu*)7697 *galE*15 *galK*16 *rpsL*(*StrR*) *endA1 nupG*, cloning host for analyzing the Sed-1 β-lactamase production
BL21(DE3)	*E. coli* (F^−^ *ompT hsd*SB(rB^−^ mB^−^) *gal dcm* (DE3)), cloning host for analyzing enzyme kinetic
Plasmids	
pCR2807	pCR-Blunt II-TOPO containing *bla*_sed-1_ and *sedR* from NR2807 amplified using Sed-1down and SedRdown
pCR2807ΔSedR	pCR-Blunt II-TOPO containing *bla*_sed-1_ from NR2807 amplified using Sed-1down and SedRUpR
pCR51115	pCR-Blunt II-TOPO containing *bla*_sed-1_ and *sedR* from pCR51115 amplified using Sed-1down and SedRdown
pCR51115ΔSedR	pCR-Blunt II-TOPO containing *bla*_sed-1_ from pCR51115 amplified using Sed-1down and SedRUpR
pCR4573	pCR-Blunt II-TOPO containing *bla*_sed-1_ and *sedR* from NR4573 amplified using Sed-1down and SedRdown
pAmpR135D	pMW219 containing *ampR* fragment of wild type (AmpR135Asp) obtained from [12]
pAmpR135A	pMW219 containing *ampR* fragment of mutant (AmpR135Ala) obtained from [12]
pCR-Blunt II-TOPO	Cloning vector purchased from Thermo Fisher Scientific, Km ^r^ Zeo ^r^
pMW219	Cloning vector purchased from Nippon Gene, Km ^r^
pET-28a (+)	Protein expression vector purchased from Novagen, Km ^r^

^a^ Rif ^r^, rifampin resistant; Km ^r^, Kanamycin resistant; Zeo ^r^, Zeocin resistant.

## Data Availability

Data will be made available on request.

## Supplementary Materials

The following supporting information can be downloaded at: https://www.mdpi.com/article/10.3390/antibiotics14080823/s1, Figure S1: Amino acid sequence alignment of the N-terminal region of AmpR and SedR (between amino acids 1 and 140); Table S1: Characteristics of inducible AmpC, derepressed AmpC, and Sed-1 producers; Table S2: Glossary of technical terms; Table S3: Primers used in the present study.

## Abbreviations

The following abbreviations are used in this manuscript:

3GC	Third-generation cephalosporins
CFU	Colony-forming units
ESBL	Extended-spectrum β-lactamase
LB	Luria–Bertani
