# Supplementary material for: Unique Regulation of Sed-1 β-Lactamase in Citrobacter sedlakii: Insights on Resistance to Third-Generation Cephalosporin
Source: Antibiotics (Basel). 2025 Aug 12;14(8):823. doi: 10.3390/antibiotics14080823 (PMC12382899; doi:10.3390/antibiotics14080823)
Supplement: Supplementary file 1 [file antibiotics-14-00823-s001.zip › antibiotics-3753595-supplementary.pdf]

Supplementary material

Unique Regulation of Sed-1 β-Lactamase in *Citrobacter sedlakii*: Insights on Resistance to Third-Generation Cephalosporin

Mako Watanabe [1,†], Ryuichi Nakano [1,*,†], Keizo Yamamoto [2], Akiyo Nakano [1], Yuki Suzuki [1], Kai Saito [1], Satoko Nakashima [3], Kentaro Endo [3], Kazuya Narita [3] and Hisakazu Yano [1]

[1] Department of Microbiology and Infectious Diseases, Nara Medical University, 840 Shijo-cho, Kashihara 6348521, Nara, Japan
[2] Department of Chemistry, Nara Medical University, 88 Shijo-cho, Kashihara 6340813, Nara, Japan
[3] Division of Central Clinical Laboratory, Iwate Medical University Hospital, 1-1-1 Idaidori, Yahaba-cho, Shiwa-gun 0283694, Iwate, Japan
* Correspondence: rnakano@naramed-u.ac.jp
† These authors contributed equally to this work.

Contents:

Supplementary Figure S1. Amino acid sequence alignment of the N-terminal region of AmpR and SedR (between amino acids 1 and 140).

Supplementary Table S1. Characteristics of inducible AmpC, derepressed AmpC, and Sed-1 producers.

Supplementary Table S2. Glossary of Technical Terms.

Supplementary Table S3. Primers used in the present study.

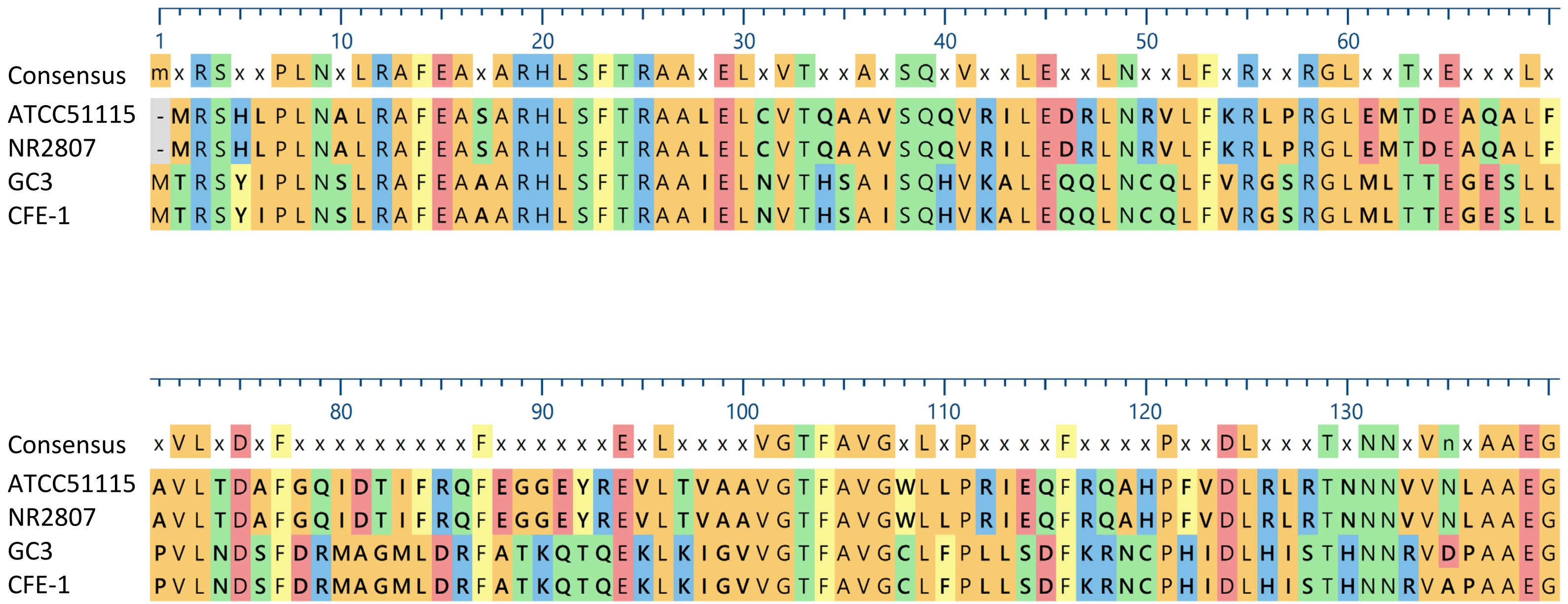

Supplementary Figure S1. Amino acid sequence alignment of the N-terminal region of AmpR and SedR (between amino acids 1 and 140). The red box highlights the amino acid residues at position 135. AmpR of *C. freundii* GC3 (inducible AmpC) and CFE-1 (derepressed AmpC) presents Asp and Ala at position 135, respectively. SedR of *C. sedlakii* ATCC51115 and NR2807 contains Asn at position 135.

Supplementary Table S1. Characteristics of inducible AmpC, derepressed AmpC, and Sed-1 producers.

β-Lactamase	Species	Strains	Transcriptional regulator	Mechanism of expression	MIC (μg/mL)[b]			Relative β-Lactamase activity (U/mg protein)			Reference or source
					PIP	CTX	CTX /CLA[c]	Basal	Induced	Induced/ basal	
Inducible AmpC	*C. freundii*	OS60	Wild-type AmpR (135Ala)	Inducible repressor	4	<0.25	NT	0.03	0.59	19.7	(10)
Derepressed AmpC (CFE-1)[a]	*E. coli*	KU6400	Mutated AmpR (135Asp)	Constitutive activator	>256	64	64	7.7	7.9	1.03	(34)
Sed-1	*C. sedlakii*	NR2807	SedR	Semi-inducible activator	256	32	0.5	4.62	5.37	1.16	This study
Sed-1	*C. sedlakii*	ATCC51115	SedR	Semi-inducible activator	8	0.5	0.25	0.17	0.40	2.31	This study

[a] CFE-1 is originated from *C. freundii ampC-ampR*

[b] Antibiotics: PIP, piperacillin; CTX, cefotaxime; CLA, clavulanic acid; NT, Not tested.

[c] MICs were determined in the presence of clavulanic acid (5 μg/mL).

Supplementary Table S2. Glossary of Technical Terms.

Term	Definition
**3GC**	Third-generation cephalosporins, a class of β-lactam antibiotics commonly used to treat Gram-negative bacterial infections.
**AmpC**	Class C β-lactamase, often chromosomally encoded and inducible, conferring resistance to cephamycins and other β-lactams.
**AmpD**	A cytosolic N-acetylmuramyl-L-alanine amidase involved in the regulation of AmpC β-lactamase expression by modulating the muropeptide pool.
**AmpR**	A LysR-type transcriptional regulator that controls the expression of AmpC; acts as a repressor or activator depending on the presence of inducers.
**Asp135Asn / AmpR135A**	A mutant form of AmpR with the substitution of Aspartic acid at position 135 to Asparagine or Alanine, leading to constitutive activation of *ampC*.
**Derepressed**	A regulatory state in which β-lactamase expression is constitutively high due to mutations in regulatory genes like *ampR* or *ampD*.
**ESBL (Extended-spectrum β-lactamase)**	Enzymes that confer resistance to penicillins and extended-spectrum cephalosporins (such as 3GCs), but are inhibited by clavulanic acid.
**Inducible**	A β-lactamase expression profile in which enzyme production is low under basal conditions but increases upon exposure to β-lactam antibiotics.
***k*cat/*K*m**	A measure of catalytic efficiency in enzyme kinetics; higher values indicate better enzyme-substrate interaction and turnover.
**MALDI-TOF MS**	Matrix-Assisted Laser Desorption/Ionization Time-of-Flight Mass Spectrometry, a technique used for rapid bacterial species identification.
**MIC (Minimum Inhibitory Concentration)**	The lowest concentration of an antibiotic that inhibits visible growth of a microorganism.
**Omega loop**	A conserved structural region (residues ~161–179) in class A β-lactamases that is important for substrate recognition and catalysis.

**pAmpR135D / pAmpR135A**	Plasmids encoding wild-type or mutant AmpR proteins, respectively, used to study regulatory effects on β-lactamase expression.
**pCR2807 / pCR51115**	Recombinant plasmids carrying *bla*$_{\text{Sed-1}}$ and regulatory genes used in transformation experiments.
**Sed-1**	A class A β-lactamase encoded by *C. sedlakii*, responsible for hydrolyzing penicillins and narrow-spectrum cephalosporins.
**SedR**	A LysR-type transcriptional regulator gene upstream of *bla*$_{\text{Sed-1}}$, involved in regulation of Sed-1 expression.
**Semi-inducible**	A regulatory profile in which β-lactamase expression is moderately enhanced upon induction, but not to the level of fully inducible systems.
**ST (Sequence Type)**	A designation based on multilocus sequence typing (MLST), used for strain typing of bacterial species.
**ANI (Average Nucleotide Identity)**	A genomic similarity index used for bacterial species delineation, generally >95–96% indicates the same species.

Supplementary Table S3. Primers used in the present study.

Primer	Sequence (5′→3′) [a]	Purpose	Reference or source
Sed-1down	TGTCTGCGCAGGGTTCTGTTC	Cloning	This study
SedRdown	TGGTACGCTGATCCCCGAAC	Cloning	This study
SedRUpR	CGGCGGGATGCGACAGT	Cloning	This study
Sed-1F-atg	CTTAAAGAACGGTTTCGCCAGAC	Enzyme kinetic analysis	This study
Sed-1R+BamHI	atatGGATCCTTACTTTCCTTCCGTCAC	Enzyme kinetic analysis	This study
Sed-1 CsA S	GCGCTGATTAATACCGC	DNA sequencing of *bla*$_{\text{Sed-1}}$	(17)
Sed-1 CsA AS	GCATCCTGCTGTGGCTGT	DNA sequencing of *bla*$_{\text{Sed-1}}$	(17)
SedRf	AGAGGGCTGGAGATGACGGATGAA	DNA sequencing of *sedR*	This study
SedRr	AGATTTCCATTGCCGGGGTCACTG	DNA sequencing of *sedR*	This study
AmpDf	ATGTTGTTAGACAAGGGCTG	DNA sequencing of *ampD*	This study
AmpDr	TCATGTCATCTCCTTATCTGAC	DNA sequencing of *ampD*	This study

[a] Underlined letters indicate *Bam*HI restriction recognition sites.
